# Kinase Hog1 and Adr1 Opposingly Regulate Haploid Cell Morphology by Controlling Vacuole Size in *Sporisorium scitamineum*

**DOI:** 10.3390/jof8080865

**Published:** 2022-08-17

**Authors:** Enping Cai, Meixin Yan, Xian Sun, Rong Zeng, Wenqiang Zheng, Yizhen Deng, Zide Jiang, Changqing Chang

**Affiliations:** 1 Integrate Microbiology Research Center, College of Plant Protection, South China Agricultural University, Guangzhou 510642, China; 2 Guangdong Laboratory for Lingnan Modern Agriculture, Guangzhou 510642, China; 3 Sugarcane Research Institute, Guangxi Academy of Agricultural Sciences, Nanning 530000, China

**Keywords:** *Sporisorium scitamineum*, MAP kinase Hog1, cAMP/PKA, cell wall permeability

## Abstract

Morphogenesis is a strictly regulated efficient system in eukaryotes for adapting to environmental changes. However, the morphogenesis regulatory mechanism in smut fungi is not clear. This study reports a relationship between MAP kinase Hog1 and cAMP-dependent protein kinase A catalytic subunit (Adr1) for the morphological regulation in the sugarcane pathogen *Sporisorium scitamineum*. The results demonstrated that MAP kinase Hog1 and cAMP/PKA signaling pathways are essential for the morphological development of *S. scitamineum*. Interestingly, MAP kinase Hog1 and cAMP/PKA signaling pathways’ defective mutants exhibit an opposite morphological phenotype. The morphology of cAMP/PKA defective mutants is recovered by deleting the *SsHOG1* gene. However, MAP kinase Hog1 and cAMP-dependent protein kinase catalytic subunit Adr1 do not interfere with each other. Further investigations showed that kinase Hog1 and Adr1 antagonistically regulates the vacuolar size, which contributes to the cell size and determines the cellular elongation rates. Kinase Hog1 and Adr1 also antagonistically balanced the cell wall integrity and permeability. Taken together, kinase Hog1- and Adr1-based opposing morphogenesis regulation of *S. scitamineum* by controlling the vacuolar size and cell wall permeability is established during the study.

## 1. Introduction

Several dimorphous fungi (animal and plant pathogens) respond to environmental conditions by changing budding growth and morphologies [1,2,3]. Morphogenetic regulation has been extensively studied including cAMP/PKA (G-protein, adenylyl cyclase, and protein kinase A) and mitogen-activated protein kinase (MAPK) pathways [4]. MAPK and cAMP/PKA signaling pathways regulate physiological processes, such as stress response, morphogenesis, polarized cell growth, mating, and virulence [5,6,7,8]. *Saccharomyces cerevisiae* cell size generally increased in response to cAMP addition [9]. MAP kinase Hog1 also played a key role in cellular responses to external stimuli and regulated morphogenesis and stress responses [10,11,12,13]. *S. cerevisiae hog1*Δ mutant cells have exhibited larger shape, abnormal bud-like extensions, and complex structure under hypertonic stress [12]. In *Cryptococcus neoformans*, mutants lacking the adenylyl cyclase Cac1 could not form titan cells, which reveals cAMP/PKA pathway involvement in the titan cells’ formation [6,14,15]. The regulation of melanin and capsule production by the opposite phenotype of Hog1 and cAMP/PKA pathways in *C. neoformans* serotype A has been reported [16]. Disruption of the cAMP/PKA genes resulted in a constitutive pseudohypha phenotype in *Ustilago maydis* [1,17,18]. *Sporisorium scitamineum* was a basidiomycetous bipolar fungus of sugarcane smut disease [19,20]. MAP kinase Hog1 and cAMP/PKA signaling pathways were known to participate in virulence and mating/filamentation of *S. scitamineum* [21,22]. However, its role in haploid cell morphogenesis remains unknown.

Signal transduction is crucial for fungi and fungal MAPK pathways might interact with other signal transduction pathways during the morphogenetic process [5]. The antagonistic role of cAMP/PKA and Kpp2-MAPK pathways during morphogenesis has also been reported in *U. maydis* [23]. The cAMP-dependent protein kinase catalytic subunit Tpk1 was required in *C. albicans* for cell wall integrity and drug tolerance [24]. Contrarily, the deletion of the *HOG1* gene conferred resistance to the cell wall perturbing agent Congo Red or Calcofluor White [11,25]. The Hog1-MAPK pathway was supposed to coordinate the chitin synthesis with other pathways [26]. Oppositely, the deletion of the *HOG1* gene slightly increased the cell wall sensitivity to Congo Red or Calcofluor White in *S. scitamineum* during our previous study [27]. Similarly, the sakA (Hog1 orthologs) defective mutant was more sensitive to cell wall damaging agents in *Aspergillus fumigatus* [28].

The role of vacuoles in eukaryotes was important during cellular biochemical pathways including cellular homeostasis, nutrient storage, protein degradation, and growth regulation through the cell cycle and death [29,30,31,32,33]. Vacuoles were the largest plant cell organelles occupying 30–80% of the cell volume and their size increased with cell growth [34]. Hence, the vacuole size determined the cell elongation rates and size [34,35,36,37]. Contrarily, inhibition of vacuolar expansion restricted plant cell growth [34,36]. The characteristics of fungal vacuoles were similar to plant and mammalian lysosomes [31]. Different mechanisms regulate the size and shape of vacuoles [38]. Defective mutants in vacuole (*vac8*Δ and *vac17*Δ) inheritance depicted the increased vacuole scaling with replicative cell age [39]. Cyclin-dependent kinase Pho85/CDK5 could also activate the vacuolar signaling pathway for initiating rapid protective mechanisms against hyperosmotic stress before long-term adaptation through Hog1 [40].

The interrelating role of the signal pathway is important in fungi. However, the interlinking mechanism between Hog1-MAPK and cAMP/PKA signaling pathways is unknown in *S. scitamineum*. This study reveals that MAP kinase Hog1 and cAMP-dependent protein kinase catalytic subunit Adr1 antagonistically regulated the morphological alterations in *S. scitamineum*. However, SsHog1 and SsAdr1 did not interfere with each other. Further investigations demonstrated that SsHog1 and SsAdr1 antagonistically regulated the vacuolar size in *S. scitamineum* by balancing cell wall integrity and permeability. In short, the results suggest that SsHog1 and SsAdr1 maintained the *S. scitamineum* morphology by mediating the vacuole size and cell wall integrity and permeability.

## 2. Materials and Methods

### 2.1. Strains and Growth Conditions

The wild-type *MAT-1* of *S. scitamineum* was isolated and identified from teliospores of sugarcane smut by Yan [41]. Details of strains are shown in Table 1. The haploid sporidia was allowed to grow in Yeast Extraction-Peptone-Sucrose medium (YePS; pH 6.5) at 28 °C, 200 rpm for 24 h. For cell wall perturbing agent assessment, *MAT-1*, *ss1adr1*Δ, *ss1hog1*Δ, and *ss1hog1*Δ*adr1*Δ haploid sporidia were allowed to grow till O.D. 600 = 1.0 (a spectrophotometer was used to measure the absorbance of haploid sporidia at 600 nm), and then 1.5 μL of sample was inoculated on YePSA medium with or without 1.0 mg/mL Congo Red (Sigma-Aldrich (Shanghai) Trading Co.Ltd, Shanghai, China) or 0.05 mg/mL Calcofluor White (Sigma-Aldrich (Shanghai) Trading Co.Ltd, Shanghai, China).

### 2.2. Strains Construction

Deletion of mutants was constructed with polyethylene glycol-mediated protoplast transformation methods as previously described [43]. The target gene was replaced by the *Zeocin^R^* (*ZEO^R^*) resistance cassette. Transformants were selected with 100 g/mL Zeocin (Invitrogen, Carlsbad, CA, USA) and were identified by PCR using genomic DNA of *MAT-1*, *ss1hog1*Δ*gpa3*Δ, *ss1hog1*Δ*uac1*Δ, and *ss1hog1*Δ*adr1*Δ as a template and the following primers: SsGPA3-inside-F/SsGPA3-inside-R, SsGPA3-outside-F/SsGPA3-outside-R, SsUAC1-inside-F/SsUAC1-inside-R, SsUAC1-outside-F/SsUAC1-outside-R, SsADR1-inside-F/SsADR1-inside-R, and SsADR1-outside-F/SsADR1-outside-R, individually. The primer sequence is shown in Table S1. In this study, transformants were constructed in the *ss1hog1*Δ background.

### 2.3. Nucleic Acid Related Manipulation

Genomic DNA of *MAT-1*, *ss1hog1*Δ, *ss1hog1*Δ*gpa3*Δ, *ss1hog1*Δ*uac1*Δ, and *ss1hog1*Δ*adr1*Δ strains were isolated by following the SDS-based DNA extraction method [44]. PCR amplification was performed using the locus-specific primers (listed in Table S1) to confirm the replacement of targeted genes with the *ZEO^R^* selection marker. For Southern blot analysis, Genomic DNA of *MAT-1*, *ss1hog1*Δ, *ss1hog1*Δ*gpa3*Δ, *ss1hog1*Δ*uac1*Δ, and *ss1hog1*Δ*adr1*Δ, and pDAN vector were digested with the restriction enzyme HindIII and the *ZEO^R^* sequence serves as the probe. For total RNA extraction, the fresh haploid was cultured in YePSA medium at 28 °C for 3 days, and then total RNA was isolated with RNeasy Mini Kit (QIAGEN, Hilden, Germany) following the established protocol [41]. For cDNA synthesis, the total RNA was used to synthesize the cDNA by HiScript^®^ II 1st Strand cDNA Synthesis Kit (Vazyme, Nanjing, China). The cDNA was performed by RT-qPCR analysis with Fast SYBR™ Green Master Mix (ThermoFisher Scientific, Carlsbad, CA, USA). The *SsGPA3-*, *SsUAC1-*, *SsADR1-*, *SsHOG1-*, wall integrity- and permeability-associated genes’ expression level was calculated with the -ΔΔCt method [45] and used cytoskeletal protein gene *GAPDH* as internal control. The experiment was conducted in triplicate and for three independent biological replicates.

### 2.4. SsHog1 and SsAdr1 Phosphorylation Assays

The fresh haploid sporidia was cultured on YEPSA for 24 h and then the total protein was extracted as previously described [21]. Phosphorylated SsHog1 or SsAdr1 was detected by Western blot analysis with the primary antibody Phospho-p38 MAPK (Cell Signaling Technology, Boston, MA, USA) or Phospho-PKA C (Cell Signaling Technology, Boston, MA, USA). Total levels of SsHog1 or SsAdr1 were detected by probing with an anti-Hog1 or anti-Adr1 antibody (Genecreate Biological Engineering Company, Wuhan, China). Blot signals were displayed with the enhanced chemiluminescence (BIO-RAD, Hercules, CA, USA) after binding of an Anti-Rabbit IgG–Peroxidase secondary antibody (Sigma, Louis, MO, USA).

### 2.5. Neutral Red Stained and Microscopy

The fresh haploid sporidia were allowed to grow in YePS medium at 28 °C, 200 rpm until O.D. 600 = 1.0, and then were treated with Neutral Red. An amount of 5 μL of the sample was mounted on the slide and observed under Leica DMI8 Inverted Fluorescence Microscope with Leica DFC450 camera (Leica, Vizsla, Germany), using Leica Application Suite (LAS) X software to take picture (Scale bar = 10 μm).

### 2.6. Flow Cytometry Analysis

*MAT-1*, *ss1adr1*Δ, *ss1hog1*Δ, and *ss1hog1*Δ*adr1*Δ haploid sporidia were allowed to grow till O.D. 600 = 1.0, and then were treated with FITC-Dextran 70,000 (1 μM) for 5 min. Then, the haploid sporidia were washed with 1 × PBS twice and re-suspended with 1 × PBS. Flow cytometry (Beckman Coulter, Inc. Pasadena, CA, USA) analysis was performed using the fluorescein isothiocyanate (FITC) filter. The sample was independently performed with three independent biological repeats, each of which contained two replications. Data analysis was performed using the FlowJo 10 software.

### 2.7. Statistical Analysis

Data were expressed as mean ± standard error (SE). Differences among different treatments were analyzed using Student’s *t*-test. The histogram was generated using GraphPad Prism 5 software.

## 3. Results

### 3.1. cAMP/PKA Kinase SsAdr1 Is a Key Factor in Controlling Haploid Cell Size of S. scitamineum

**Figure 1:**
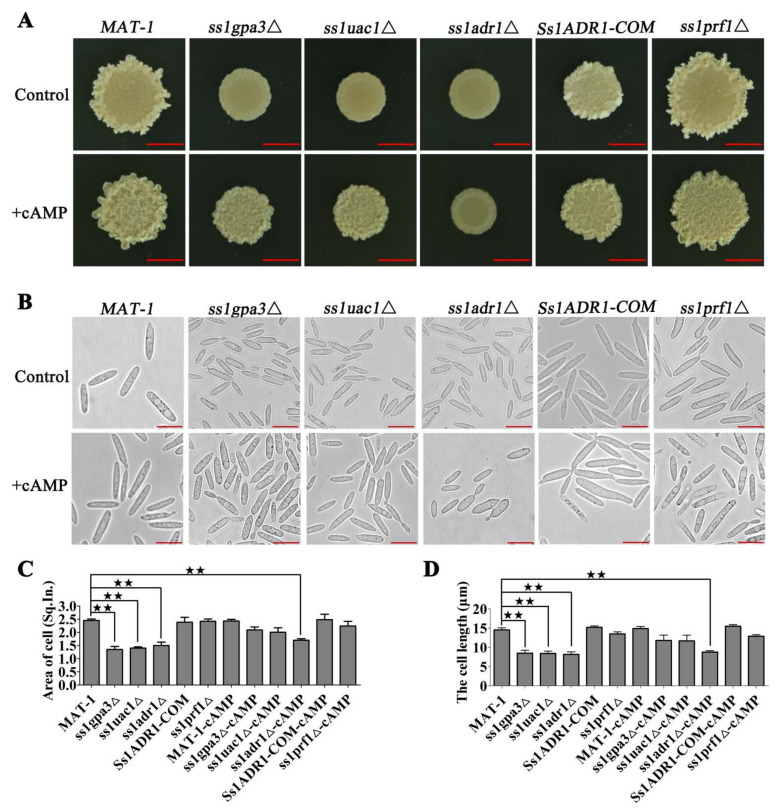
Effects of cAMP/PKA signaling pathways on morphology of S. scitamineum. (**A**) The colony morphology of *MAT-1*, *ss1gpa3*Δ, *ss1uac1*Δ, *ss1adr1*Δ, *Ss1ADR1-COM*, and *ss1prf1*Δ strains. *MAT-1*, *ss1gpa3*Δ, *ss1uac1*Δ, *ss1adr1*Δ, *Ss1ADR1-COM*, and *ss1prf1*Δ haploid sporidia were cultured under YePSA medium with or without exogenetic cAMP (5 mM). Images were taken 4 days after cultivation. Scale bar = 5.0 mm. (**B**) The microscopic morphology of wild-type *MAT-1*, *ss1gpa3*Δ, *ss1uac1*Δ, *ss1adr1*Δ, *Ss1ADR1-COM*, and *ss1prf1*Δ strains. *MAT-1*, *ss1gpa3*Δ, *ss1uac1*Δ, *ss1adr1*Δ, *Ss1ADR1-COM*, and *ss1prf1*Δ haploid sporidia were allowed to grow till O.D. 600 = 1.0, and treated with or without exogenetic cAMP (5 mM) under YePSA medium at 28 °C for 24 h. Images were taken by Leica DMI8 Inverted Fluorescence Microscope. Scale bar = 10 µm. (**C**) The cell areas of *MAT-1*, *ss1gpa3*Δ, *ss1uac1*Δ, *ss1adr1*Δ, *Ss1ADR1-COM*, and *ss1prf1*Δ strains were measured. *MAT-1*, *ss1gpa3*Δ, *ss1uac1*Δ, *ss1adr1*Δ, *Ss1ADR1-COM*, and *ss1prf1*Δ haploid sporidia were allowed to grow till O.D. 600 = 1.0 with or without exogenetic cAMP (5 mM), and then images were taken by Leica DMI8 Inverted Fluorescence Microscope. Adobe Photoshop software was used to calculate the pixels of each cell. One square inch (Sq. In.) is 72 by 72 pixels. Bar chart depicts statistical difference between the mean values (^★★^ *p* < 0.01). The sample was independently performed with three independent biological repeats, each of which contained twenty replications. (**D**) The cell lengths of *MAT-1*, *ss1gpa3*Δ, *ss1uac1*Δ, *ss1adr1*Δ, *Ss1ADR1-COM*, and *ss1prf1*Δ strains were measured. *MAT-1*, *ss1gpa3*Δ, *ss1uac1*Δ, *ss1adr1*Δ, *Ss1ADR1-COM*, and *ss1prf1*Δ haploid sporidia were allowed to grow till O.D. 600 = 1.0 with or without exogenetic cAMP (5 mM), and then images were taken by Leica DMI8 Inverted Fluorescence Microscope and the length of the cell was measured. The unit is µm. Bar chart depicts statistical difference between the mean values (^★★^ *p* < 0.01). The sample was independently performed with three independent biological repeats, each of which contained twenty replications.

The impact of the cAMP/PKA signaling pathway on morphogenesis was analyzed by culturing *SsADR1* and its upstream genes’ (*SsGPA3* and *SsUAC1*) deletion mutants (details of strains are shown in Table 1) on the YePSA medium. The results showed that the morphogenesis of the cAMP/PKA defective mutant colonies was changed to the glossy surface phenotype. This phenomenon could be partially restored through the exogenous addition of cAMP in *ss1gpa3*Δ and *ss1uac1*Δ mutants as compared to wild type (Figure 1A). However, *ss1adr1*Δ mutants still exhibited a distinct glossy surface, even after cAMP treatment (Figure 1A). The disruption of the *SsPRF1* gene [42] encoding the pheromone response factor and located downstream of *SsADR1* shared a similar normal morphology with the wild type (Figure 1A). The area and length of the haploid sporidia were also measured, which revealed significantly smaller cell size in cAMP/PKA defective mutants (*ss1gpa3*Δ, *ss1uac1*Δ, *ss1adr1*Δ) as compared to the wild-type and complementary strains (Figure 1B–D). These results indicated that the cell size of cAMP/PKA defective mutants was diminished by reducing the cAMP signal and was partially restored by the exogenous addition of cAMP in *ss1gpa3*Δ and *ss1uac1*Δ mutants except in the *ss1adr1*Δ mutant (Figure 1B–D). Overall, the results indicated the necessity of the cAMP/PKA pathway in morphogenesis whereas SsAdr1 played a key role in the *S. scitamineum* morphological regulation.

### 3.2. MAP Kinase SsHog1 Is Required for Maintaining Haploid Cell Morphology of S. scitamineum

**Figure 2:**
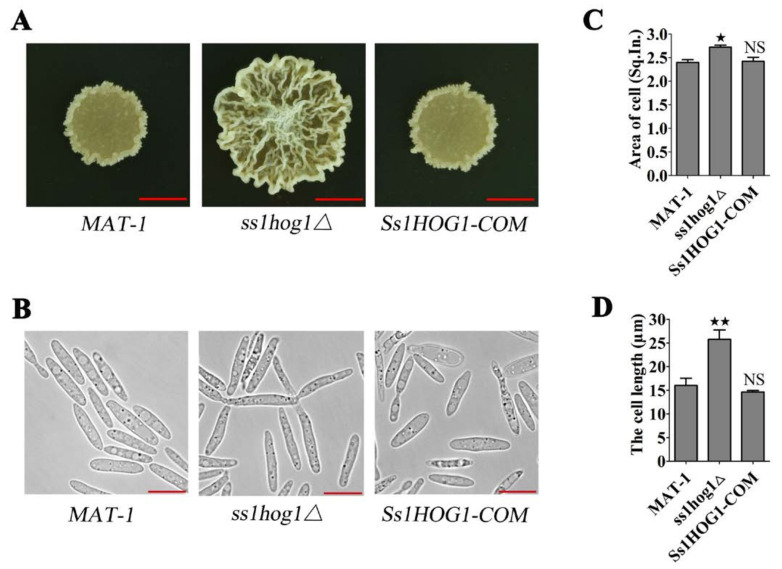
Effects of SsHog1 on morphology of *S. scitamineum*. (**A**) The colony morphology of *MAT-1*, *ss1hog1*Δ, and *Ss1HOG1-COM* strains. The fresh haploid sporidia were spotted onto YePSA medium at 28 °C. Images were taken 4 days after cultivation. Scale bar = 5.0 mm. (**B**) The microscopic morphology of *MAT-1*, *ss1hog1*Δ, and *Ss1HOG1-COM* strains. *MAT-1*, *ss1hog1*Δ, and *Ss1HOG1-COM* haploid sporidia were allowed to grow till O.D. 600 = 1.0, and then 5 μL of the sample was mounted on the slide and observed. Images were taken by Leica DMI8 Inverted Fluorescence Microscope. Scale bar = 10 µm. (**C**) The cell areas of *MAT-1*, *ss1hog1*Δ, and *Ss1HOG1-COM* strains were measured. *MAT-1*, *ss1hog1*Δ, and *Ss1HOG1-COM* haploid sporidia were allowed to grow till O.D. 600 = 1.0, and then images were taken by Leica DMI8 Inverted Fluorescence Microscope. Adobe Photoshop software was used to calculate the pixels of each cell. One square inch (Sq. In.) is 72 by 72 pixels. Bar chart depicts statistical difference between the mean values (^★^ *p* < 0.05). NS denotes a not statistically significant difference (*p* < 0.05). The sample was independently performed with three independent biological repeats, each of which contained twenty replications. (**D**) The cell lengths of *MAT-1*, *ss1hog1*Δ, and *Ss1HOG1-COM* strains were measured. The fresh haploid sporidia were allowed to grow till O.D. 600 = 1.0, and then images were taken by Leica DMI8 Inverted Fluorescence Microscope and the length of the cell was measured. The unit is µm. Bar chart depicts statistical difference between the mean values (^★★^ *p* < 0.01). NS denotes a not statistically significant difference (*p* < 0.05). The sample was independently performed with three independent biological repeats, each of which contained twenty replications.

The *hog1*Δ mutant cells exhibited an irregular and larger cell morphology in *S. cerevisiae* under hypertonic stress [12]. However, the cell morphology of *ss1hog1*Δ mutants (with or without hypertonic stress) in *S. scitamineum* were not significantly different (unshown). Nevertheless, *ss1hog1*Δ mutant colonies appeared bigger with a folded surface in YePSA medium, compared with the wild-type and *Ss1HOG1-COM* strains (Figure 2A). Furthermore, the *ss1hog1*Δ mutant displayed a larger cell area and longer cell length, in comparison to the wild-type and *Ss1HOG1-COM* strains (Figure 2B–D). In short, SsHog1 maintained a stable *S. scitamineum* morphology.

### 3.3. The Deletions of SsADR1 and SsHOG1 Do Not Affect Expression of Each Other in S. scitamineum

**Figure 3:**
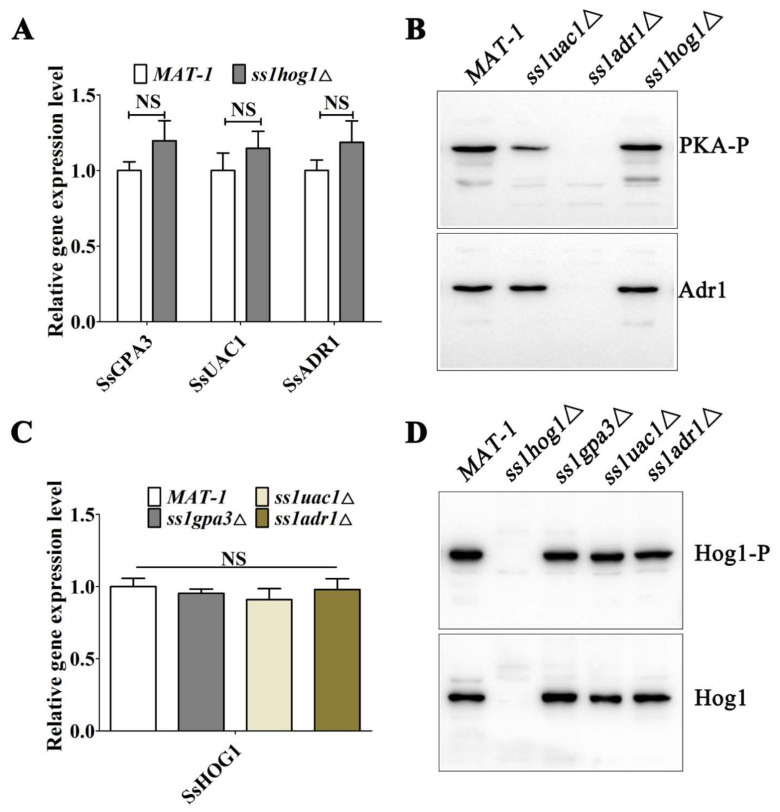
The relationship between MAP kinase Hog1 and cAMP/PKA signaling pathways. (**A**) Transcriptional profile of cAMP/PKA-pathway-related genes in *MAT-1* and *ss1hog1*Δ. The transcription levels of genes *SsGPA3*, *SsUAC1*, and *SsADR1* in the cAMP/PKA pathway were detected in the *MAT-1* and *ss1hog1*Δ. NS denotes a not statistically significant difference (*p* < 0.05). The sample was independently performed with three independent biological repeats, each of which contained two replications. (**B**) The phosphorylated levels of PKA in *MAT-1*, *ss1adr1*Δ, and *ss1hog1*Δ strains. Total protein was extracted from the fresh haploid sporidia under YePSA medium. The phosphorylated PKA was detected with the primary antibody Phospho-PKA C and the total level of SsAdr1 was determined with anti-Adr1 antibody by Western blot analysis. (**C**) Transcriptional profile of *SsHOG1* was detected in *MAT-1*, *ss1gpa3*Δ, *ss1uac1*Δ, and *ss1adr1*Δ. NS denotes a not statistically significant difference (*p* < 0.05). The sample was independently performed with three independent biological repeats, each of which contained two replications. (**D**) The phosphorylated levels of SsHog1 in *MAT-1*, *ss1hog1*Δ, *ss1gpa3*Δ, *ss1uac1*Δ, and *ss1adr1*Δ strains. Total protein was extracted from the fresh haploid sporidia under YePSA medium. The phosphorylated Hog1 was detected with the primary antibody Phospho-p38 MAPK (Thr180/Tyr182) and the total level of SsHog1 was determined with anti-Hog1 antibody by Western blot analysis.

Based on the above-mentioned results, the potential molecular interaction between SsHog1 and the cAMP/PKA pathway was investigated by analyzing the mRNA transcription and protein phosphorylation levels in *ss1hog1*Δ and cAMP/PKA defective mutants. The results demonstrated that the *SsGPA3*, *SsUAC1*, and *SsADR1* gene transcription levels were not significantly different than wild-type and *ss1hog1*Δ mutants (Figure 3A). Moreover, PKA phosphorylation levels in *ss1hog1*Δ mutants were almost similar to wild-type *MAT-1* (Figure 3B). Similarly, the transcription level of the *SsHOG1* gene (Figure 3C) and the phosphorylation level of SsHog1 (Figure 3D) was not significantly different than wild-type and cAMP/PKA defective mutants. Taken together, these results suggested the lack of interference between SsHog1 and SsAdr1, and they might split for regulating the morphology.

### 3.4. Deletion of SsHOG1 Recovers the Morphology of cAMP/PKA Defective Mutants

The causes of *ss1hog1*Δ and cAMP/PKA defective mutants’ contradictory impacts on morphology. The deletion strains of the cAMP/PKA pathway under the background of *ss1hog1*Δ mutants were individually generated by homologous recombination, and named as *ss1hog1*Δ*gpa3*Δ, s*s1hog1*Δ*uac1*Δ, and *ss1hog1*Δ*adr1*Δ. Target-sequence disruption in mutants was confirmed through PCR amplification (Figure S1A,B). Detection of double-deletion mutants served as the *Zeocin* gene probe through Southern blotting (Figure S1C). The transcriptional profile confirmed the disappearance of *SsGPA3*, *SsUAC1*, and *SsADR1* genes’ expressions in double-deletion mutants, thus indicating the destruction of the original gene (Figure S1D).

**Figure 4:**
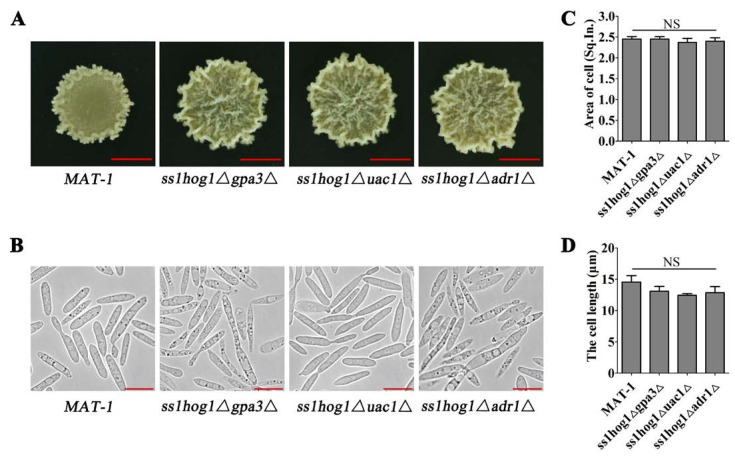
Morphology of SsHog1 and cAMP/PKA signaling pathways’ double-deletion mutants. (**A**) The colony morphology of *MAT-1*, *ss1hog1*Δ*gpa3*Δ, *ss1hog1*Δ*uac1*Δ, and *ss1hog1*Δ*adr1*Δ strains. *MAT-1*, *ss1hog1*Δ*gpa3*Δ, *ss1hog1*Δ*uac1*Δ, and *ss1hog1*Δ*adr1*Δ haploid sporidia were spotted onto YePSA medium at 28 °C. Images were taken 4 days after cultivation. Scale bar = 5.0 mm. (**B**) The microscopic morphology of *MAT-1*, *ss1hog1*Δ*gpa3*Δ, *ss1hog1*Δ*uac1*Δ, and *ss1hog1*Δ*adr1*Δ strains. *MAT-1*, *ss1hog1*Δ*gpa3*Δ, *ss1hog1*Δ*uac1*Δ, and *ss1hog1*Δ*adr1*Δ haploid sporidia were allowed to grow till O.D. 600 = 1.0, and then 5 μL of the sample was mounted on the slide and observed. Images were taken by Leica DMI8 Inverted Fluorescence Microscope. Scale bar = 10 µm. (**C**) The cell areas of *MAT-1*, *ss1hog1*Δ*gpa3*Δ, *ss1hog1*Δ*uac1*Δ, and *ss1hog1*Δ*adr1*Δ strains were measured. *MAT-1*, *ss1hog1*Δ*gpa3*Δ, *ss1hog1*Δ*uac1*Δ, and *ss1hog1*Δ*adr1*Δ haploid sporidia were allowed to grow till O.D. 600 = 1.0, and then images were taken by Leica DMI8 Inverted Fluorescence Microscope. Adobe Photoshop software was used to calculate the pixels of each cell. One square inch (Sq. In.) is 72 by 72 pixels. Bar chart depicts statistical difference between the mean values (*p* < 0.05). NS denotes a not statistically significant difference. The sample was independently performed with three independent biological repeats, each of which contained twenty replications. (**D**) The cell lengths of *MAT-1*, *ss1hog1*Δ*gpa3*Δ, *ss1hog1*Δ*uac1*Δ, and *ss1hog1*Δ*adr1*Δ strains were measured. *MAT-1*, *ss1hog1*Δ*gpa3*Δ, *ss1hog1*Δ*uac1*Δ, and *ss1hog1*Δ*adr1*Δ haploid sporidia were allowed to grow till O.D. 600 = 1.0, and the length of the cell was measured. Images were taken by Leica DMI8 Inverted Fluorescence Microscope. The unit is µm. Bar chart depicts statistical difference between the mean values (*p* < 0.05). NS denotes a not statistically significant difference. The sample was independently performed with three independent biological repeats, each of which contained twenty replications.

The glossy surface disappeared in double-deletion mutants as compared to cAMP/PKA defective mutants. The colony morphologies of *ss1hog1*Δ*gpa3*Δ, s*s1hog1*Δ*uac1*Δ, and *ss1hog1*Δ*adr1*Δ mutants were fully restored like *ss1hog1*Δ in the YePSA medium (Figure 1A and Figure 4A). However, the cell areas and cell lengths of double-deletion mutants returned to the wild type (Figure 4B–D). In short, the glossy surface of the cAMP/PKA defective mutants was recovered by deleting the *SsHOG1* gene.

### 3.5. The SsHog1 and SsAdr1 Collaborate for Haploid Cell Morphology by Controlling Vacuole Size in S. scitamineum

**Figure 5:**
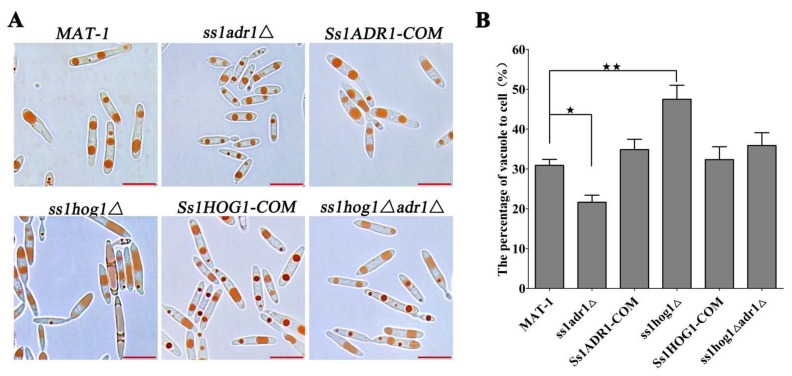
Effect of SsHog1 and SsAdr1 on vacuolar size. (**A**) Neutral Red staining in the *MAT-1*, *ss1adr1*Δ, *Ss1ADR1-COM*, *ss1hog1*Δ, *Ss1HOG1-COM*, and *ss1hog1*Δ*adr1*Δ strains. The Neutral Red was mixed with the fresh haploid sporidia, and then 5 μL of the sample was mounted on the slide and observed (Scale bar = 10 μm). (**B**) The vacuole-to-cell size scaling ratio of *MAT-1*, *ss1adr1*Δ, *Ss1ADR1-COM*, *ss1hog1*Δ, *Ss1HOG1-COM*, and *ss1hog1*Δ*adr1*Δ strains. Bar chart depicts statistical difference between the mean values (^★^ *p* < 0.05 and ^★★^ *p* < 0.01). The sample was independently performed with three independent biological repeats. For each instance *n* > 50, samples were assessed.

The vacuole size is known to correlate with cell size in plants and vacuolar expansion inhibition could restrict cell growth [34,36,46]. Therefore, the vacuoles were observed as orange-red by Neutral Red staining in *S. scitamineum* (Figure 5A). As expected, the vacuoles of the *ss1adr1*Δ mutants were diminished with a significant reduction in vacuole-to-cell size scaling ratio, compared with the wild-type and *Ss1ADR1-COM* strains (Figure 5A,B). Contrarily, the larger vacuoles and the vacuole-to-cell size scaling ratio were present in *ss1hog1*Δ mutant cells in comparison to the wild-type and *Ss1HOG1-COM* strains. Interestingly, the size of the vacuole and the ratio of vacuole-to-cell scaling in *ss1hog1*Δ*adr1*Δ mutant cells did not exhibit significantly changes as compared to the wild type (Figure 5A,B). Taken together, SsHog1 and SsAdr1 exhibited a correlation with vacuole size, which might regulate the cell morphology by influencing the vacuole size.

### 3.6. SsHog1 and SsAdr1 Regulate Cell Wall Permeability to Maintain Cell Size

The regulatory network of SsHog1 and SsAdr1 involved in morphological regulation was further explored by performing transcriptome analysis with the *ss1adr1*Δ, *ss1hog1*Δ, and wild-type sporidia grown in the YePSA medium. Differentially expressed genes (DEGs) were identified in the *ss1adr1*Δ and *ss1hog1*Δ mutants, compared to the wild-type *MAT-1* strain. Gene ontology (GO) enrichment of the DEGs is represented in Figure S2. The “transmembrane transport” (GO:0055085) and “transport activity” (GO:0005215) were found to be more significant descriptors in the *ss1hog1*Δ mutant. Interestingly, “transport activity” was also a highly significant descriptor in the *ss1adr1*Δ mutant, suggesting an important role of MAP kinase Hog1 and cAMP-dependent protein kinase catalytic subunit Adr1in transport activity.

**Figure 6:**
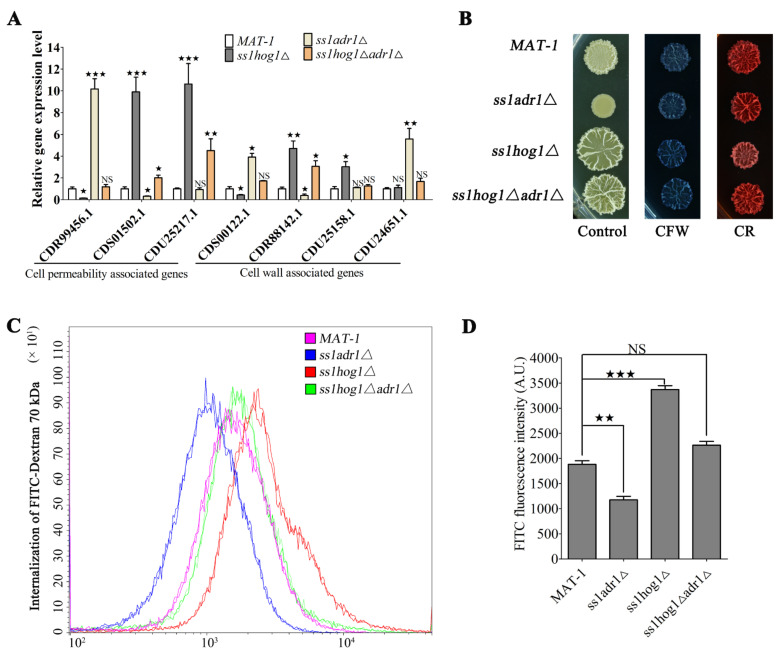
Effects of SsHog1 and SsAdr1 on morphology cell wall integrity and permeability. (**A**) Transcriptional profile of cell wall integrity- and permeability-related genes in the *MAT-1*, *ss1adr1*Δ, *ss1hog1*Δ, and *ss1hog1*Δ*adr1*Δ strains. Total RNA was extracted from the haploid sporidia which were allowed to grow on YePSA medium at 28 °C for 3 days, and then RT-qPCR was used to analyze the expression of genes related to cell wall integrity and permeability in the wild type and mutants. Relative gene expression level was calculated with the -ΔΔCt method with *GAPDH* as internal control. Bar chart depicts the statistical difference between the mean values (^★^ *p* < 0.05, ^★★^ *p* < 0.01, ^★★★^ *p* < 0.001). NS denotes a not statistically significant difference. The sample was independently performed with three independent biological repeats, each of which contained two replications. NS denotes a not statistically significant difference (*p* < 0.05). (**B**) Effects of Congo Red (CR) and Calcofluor White (CFW) on morphology of *S. scitamineum*. The fresh haploid sporidia were allowed to grow till O.D. 600 = 1.0, and then 1.5 μL of cells was spotted onto YePSA medium with or without 1.0 mg/mL CR and 0.05 mg/mL CFW. Images were taken 5 days after cultivation. (**C**) Flow cytometry analyses of FITC-Dextran 70,000 internalized in the *MAT-1*, *ss1adr1*Δ, *ss1hog1*Δ, and *ss1hog1*Δ*adr1*Δ strains. *MAT-1*, *ss1adr1*Δ, *ss1hog1*Δ, and *ss1hog1*Δ*adr1*Δ haploid sporidia were allowed to grow till O.D. 600 = 1.0 and treated with FITC-Dextran 70,000 (1 μM) for 5 min. Then, cells were washed with PBS twice and re-suspended with PBS. Flow cytometry analysis was performed using the fluorescein isothiocyanate (FITC) filter. The sample was independently performed with three independent biological repeats, each of which contained two replications. Data analysis was performed using the FlowJo 10 software. (**D**) Bar chart depicting quantification of FITC fluorescence intensity as shown in (**C**). Bar chart depicts the statistical difference between the mean values (^★★^ *p* < 0.01, ^★★★^ *p* < 0.001). NS denotes a not statistically significant difference. The sample was independently performed with three independent biological repeats, each of which contained two replications. NS denotes a not statistically significant difference (*p* < 0.05).

We next wondered whether differential expression of “transport activity” genes affected cell wall integrity and permeability. The wall integrity- and permeability-associated genes, including *CDR99456.1* (encoding an Sge1 drug-resistance protein), *CDS01502.1* (encoding a Pho8 alkaline phosphatase), *CDU25217.1* (encoding a probable purine permease), *CDS00122.1* (encoding a glucan synthase subunit), *CDR88142.1* (encoding glycosyl hydrolases), *CDU25158.1* (encoding glycosyl hydrolases), and *CDU24651.1* (encoding a chitinase), were subjected to quantitative real-time PCR (RT-qPCR) analysis. The results showed that transcriptional expression of the *CDR99456.1*, *CDS00122.1*, and *CDU24651.1* genes were significantly (*p* < 0.05) up-regulated in the *ss1adr1*Δ mutant. Contrarily, gene expressions in the *ss1hog1*Δ*adr1*Δ mutant were not significantly different from the wild type (Figure 6A). Moreover, the *CDR99456.1* and *CDS00122.1* genes were reduced in the *ss1hog1*Δ mutant (Figure 6A). The transcription level of *CDS01502.1*, *CDU25217.1*, *CDR88142.1*, and *CDU25158.1* genes were significantly (*p* < 0.05) increased in the *ss1hog1*Δ mutant and slightly down-regulated or remained undifferentiated in the *ss1adr1*Δ mutant as compared to the wild type (Figure 6A). The transcription levels of these genes decreased in the *ss1hog1*Δ*adr1*Δ mutant than *ss1hog1*Δ mutant (Figure 6A). Previously, we have reported the necessity of MAP kinase Hog1 for the cell wall integrity in *S. scitamineum* [27]. Therefore, *MAT-1*, *ss1adr1*Δ, *ss1hog1*Δ, and *ss1hog1*Δ*adr1*Δ strains were cultured on the YEPS medium containing the cell wall perturbing agent Congo Red (CR) or Calcofluor White (CFW). The results showed that the glossy surface phenotype in the *ss1adr1*Δ mutant could be fully restored by the exogenous addition of Congo Red or Calcofluor White, which returned the *ss1hog1*Δ mutant colony morphology to the level of the wild type (Figure 6B). To compare the change of permeability between these mutants and the wild type, the internalization rate of FITC-Dextran 70,000 [47] was measured in the *MAT-1*, *ss1adr1*Δ, *ss1hog1*Δ, and *ss1hog1*Δ*adr1*Δ strains followed by flow cytometry. Internalized FITC-Dextran 70,000 was mostly found in the *ss1hog1*Δ mutant whereas the *ss1adr1*Δ mutant had its lowest presence. FITC-Dextran 70,000 in the *ss1hog1Δadr1Δ* mutant was similar to the wild type (Figure 6C,D). Taken together, cell wall integrity and permeability were noted to be correlated with MAP kinase Hog1 and cAMP-dependent protein kinase catalytic subunit.

## 4. Discussion

MAPK and cAMP/PKA signaling pathways in fungi transmit the informational from the extracellular to the intracellular environment through important conservative mechanisms [5,16,48]. Morphogenesis facilitates the adaptation to environmental transformation by changing cellular dimensions [49,50]. The oversized cells exhibited slow cell division, cytoplasmic dilution, and altered transcriptome and proteome, suggesting that normal morphology maintenance is important for biological and physiological functions [51]. This study demonstrates that the SsHog1 and cAMP/PKA pathways regulate the *S. scitamineum* morphology by mediating the vacuole size, cell wall integrity, and permeability.

The role of the cAMP/PKA pathway in fungal morphology regulation has been reported. The cAMP/PKA defective mutants exhibited glossy colony morphology and smaller cellular size. This phenotype is opposite to *U. maydis*, which disrupts cAMP/PKA pathways for constitutive pseudohypha growth [1,17,18]. Furthermore, the disruption of the *SsPRF1* gene (located downstream of *SsADR1*) resulted in normal morphology similar to the wild type. The exogenous addition of cAMP partially restored the *ss1gpa3*Δ and *ss1uac1*Δ mutants’ morphology except for the *ss1adr1*Δ mutants. These results indicate that SsGpa3 and SsUac1 regulate the SsAdr1 activity by cAMP, which further regulates morphogenesis in other target proteins rather than through the transcription factor SsPrf1. Interestingly, the glossy surface phenotype in the *ss1adr1*Δ mutant was fully restored after the exogenous addition of Congo Red or Calcofluor White. These findings suggest the involvement of SsAdr1 in cell wall integrity. The cAMP/PKA pathway participation in the cell wall integrity of *C. neoformans* has been reported [52]. We hypothesized that SsAdr1 regulates the biosynthesis or degradation of glucan and/or chitin but this requires further verification. Vacuolar size alteration is also an important factor affecting cellular morphogenesis. The vacuolization contributes to cell size heterogeneity and might resolve the conflicts regarding growth control at the cellular and organ levels [34,35,36,37,53]. The vacuolar size also increases with the cell size in fungi [38]. Further investigations showed that the vacuole-to-cell scaling ratio was diminished in the *ss1adr1*Δ mutant as compared to the wild type, suggesting that a smaller vacuole-to-cell scaling ratio is more likely to cause cell shrinkage. Taken together, these data indicated that SsAdr1 plays a crucial role in the regulation of morphology.

MAPK signaling pathways are also important in morphological regulation. In this study, the *ss1hog1*Δ mutants displayed a wrinkled colony morphology and a larger cell size under the ordinary culture. These results indicate that SsHog1 negatively regulates haploid cell morphogenesis, which is similar to the negative regulation of the *HOG1* gene in *S. cerevisiae* under hypertonic stress [12]. Moreover, the large colony morphology of the *ss1hog1*Δ mutant returned to the level of the wild type after treatment with Congo Red or Calcofluor White. The reduced tolerance of *ss1hog1*Δ mutants to cell wall stress reagents might be the reason behind this phenomenon. The importance of MAP kinase Hog1 for the cell wall integrity in *S. scitamineum* has been reported [27]. This is an opposite phenotype to *C. albicans*, where deletion of the *HOG1* gene enhanced the tolerance to Congo Red and Calcofluor White [25]. In addition, transcriptional expression of the glycosyl hydrolases genes (*CDR88142.1* and *CDU25158.1*) and internalization of FITC-Dextran 70,000 were significantly (*p* < 0.05) increased in the *ss1hog1*Δ mutant as compared to the wild-type. It suggests that the change in cell wall permeability also affects morphogenesis.

What is the regulatory relationship between the Hog1-MAPK pathway and cAMP/PKA signaling pathways in the morphologies of *S. scitamineum*? To explore this question, the *ss1hog1*Δ*gpa3*Δ, *ss1hog1*Δ*uac1*Δ and *ss1hog1*Δ*adr1*Δ mutants were generated according to the *ss1hog1*Δ mutant. The results revealed that the morphology of cAMP/PKA defective mutants was recovered with the deletion of the *SsHOG1* gene, which suggests that SsHog1 might be located downstream of the cAMP/PKA signaling pathway or is a parallel pathway. These findings are similar to *C. neoformans*, where the disruption of genes (*GPA1*, *CAC1,* or *PKA1* in *hog1*Δ mutant background) restored melanin production, compared with the complete elimination of melanin formation in *gpa1*Δ, *cac1*Δ, or *pka1*Δ single mutants [16]. The abundance of ribosomal protein (RP) transcripts and the biosynthesis of ribosomes in *C. albicans* were maintained at mRNA stabilization by the dual and opposing actions of Hog1 and cAMP/PKA pathways [54]. Further investigations revealed that there were not significant changes in the transcription level of the *SsHOG1* gene and phosphorylation level of SsHog1 in cAMP/PKA defective mutants, and vice versa, suggesting that MAP kinase Hog1 and SsAdr1 did not interfere with each other in *S. scitamineum*. These results indicated that SsHog1 is not located downstream of the cAMP/PKA signaling pathway. The next question is why deletion of *SsHOG1* recovered the cAMP/PKA defective mutant morphology, even more like *ss1hog1*Δ. We think that SsHog1 and SsAdr1 antagonistically regulated morphological development by mediating vacuolar size, cell wall integrity and permeability in *S. scitamineum*. Perhaps SsHog1 might have contributed more to maintaining the normal morphology than cAMP/PKA signaling pathways, or other factors.

To conclude, the study establishes that SsHog1 and the cAMP/PKA pathways are required for a particular morphology. The data further elaborated the antagonistic morphology regulation by mediating vacuolar size, cell wall integrity, and permeability in *S. scitamineum*.

## Figures and Tables

**Table 1:** Details of strains shown in this study.

Strains	Accession Number for Protein	Genotype (Resistance)	Reference or Source
*MAT-1*	wild type	*a1, b1*	[41]
*ss1gpa3*Δ	CDU22142.1	*a1, b1* Δ*gpa3* (*HYG^R^*)	[21]
*ss1uac1*Δ	CDU22142.1	*a1, b1* Δ*uac1* (*HYG^R^*)	[21]
*ss1adr1*Δ	CDU22142.1	*a1, b1* Δ*adr1* (*HYG^R^*)	[21]
*Ss1ADR1-COM*	CDU22142.1	*a1, b1* Δ*adr1* *adr1*(*ZEO^R^*)	[21]
*ss1prf1*Δ	CDU21933.1	*a1, b1* Δ*prf1* (*HYG^R^*)	[42]
*ss1hog1*Δ	CDU21933.1	*a1, b1* Δ*hog1* (*HYG^R^*)	[27]
*Ss1HOG1-COM*	CDU21933.1	*a1, b1* Δ*hog1 hog1* (*ZEO^R^*)	[27]
*ss1hog1*Δ*gpa3*Δ	CDU22378.1	*a1, b1* Δ*hog1* Δ*gpa3* (*HYG^R^ ZEO^R^*)	This study
*ss1hog1*Δ*uac1*Δ	CDU25762.1	*a1, b1* Δ*hog1* Δ*uac1* (*HYG^R^ ZEO^R^*)	This study
*ss1hog1*Δ*adr1*Δ	CDU22361.1	*a1, b1* Δ*hog1* Δ*adr1* (*HYG^R^ ZEO^R^*)	This study

## Data Availability

All data required to understand this article are presented in the study or the Supplementary Materials. Any raw data further requested will be provided by the corresponding authors.

## Supplementary Materials

The following supporting information can be downloaded at: https://www.mdpi.com/article/10.3390/jof8080865/s1, Figure S1: Identification of mutants by PCR amplification, Southern blot, and RT-qPCR, Figure S2: GO enrichment analysis of differentially expressed genes (DEGs) in the *MAT-1*, *ss1hog1*Δ, and *ss1adr1*Δ strains, Table S1: The primers and sequences used in this study.
