# Supplementary material for: Kinase Hog1 and Adr1 Opposingly Regulate Haploid Cell Morphology by Controlling Vacuole Size in Sporisorium scitamineum
Source: J Fungi (Basel). 2022 Aug 17;8(8):865. doi: 10.3390/jof8080865 (PMC9410113; doi:10.3390/jof8080865)
Supplement: Supplementary file 1 [file jof-08-00865-s001.zip › jof-1814599-supplementary.pdf]

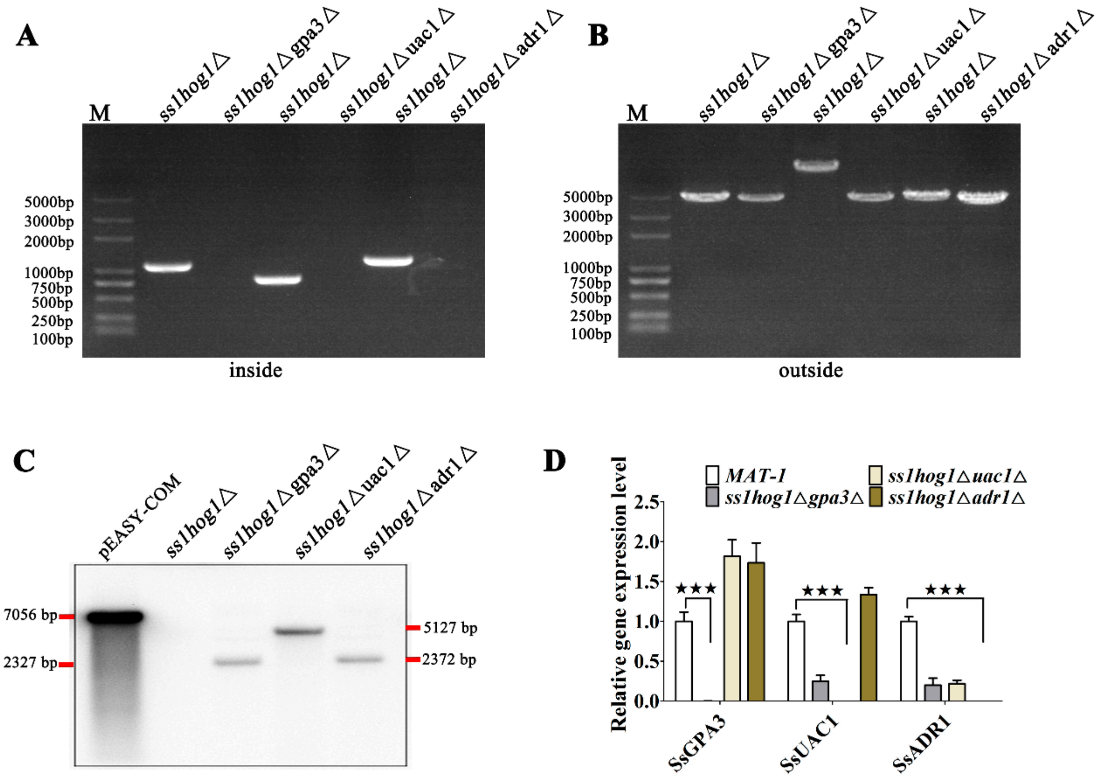

Figure S1. Identification of mutants by PCR amplification, Southern blot, and RT-qPCR. (A) and (B) PCR amplification was performed using specific primers inside-F/inside-F or outside-F/outside-F (listed in Table S1) to confirm the replacement of targeted gene with the *ZEO*^{R} selection marker. Molecular markers in bp were labeled. (C) Southern blot analysis was performed for confirming the deletion mutants. The genomic DNA of *ss1hog1*Δ, *ss1hog1*Δ*gpa3*Δ, *ss1hog1*Δ*uac1*Δ, *sss1hog1*Δ*adr1*Δ, and pEASY-COM plasmid were digested with the restriction enzyme *Hind*III at 37 °C for overnight. The *ZEO*^{R} gene was used as the probe. The 7056 bp band of the pEASY-COM plasmid served as a positive control of the experimental procedure. Probed bands of 2327 bp, 5127 bp and 2372 bp size in the *ss1hog1*Δ*gpa3*Δ, *ss1hog1*Δ*uac1*Δ, and *sss1hog1*Δ*adr1*Δ mutants confirmed the correct gene replacement events, severally. (D) RT-qPCR analysis for expression of *SsGPA3*, *SsUAC1*, and *SsADR1* genes in the wild type and mutants under sporidial growth on YePSA plate for 24 h, respectively. Relative gene expression level was calculated with -ΔΔCt method with *GAPDH* as internal control. Barchart depicts statistical difference among the mean values (★★★*p* <0.001). Mean ± S.E. are derived from three independent biological repeats, each of which contained three replications.

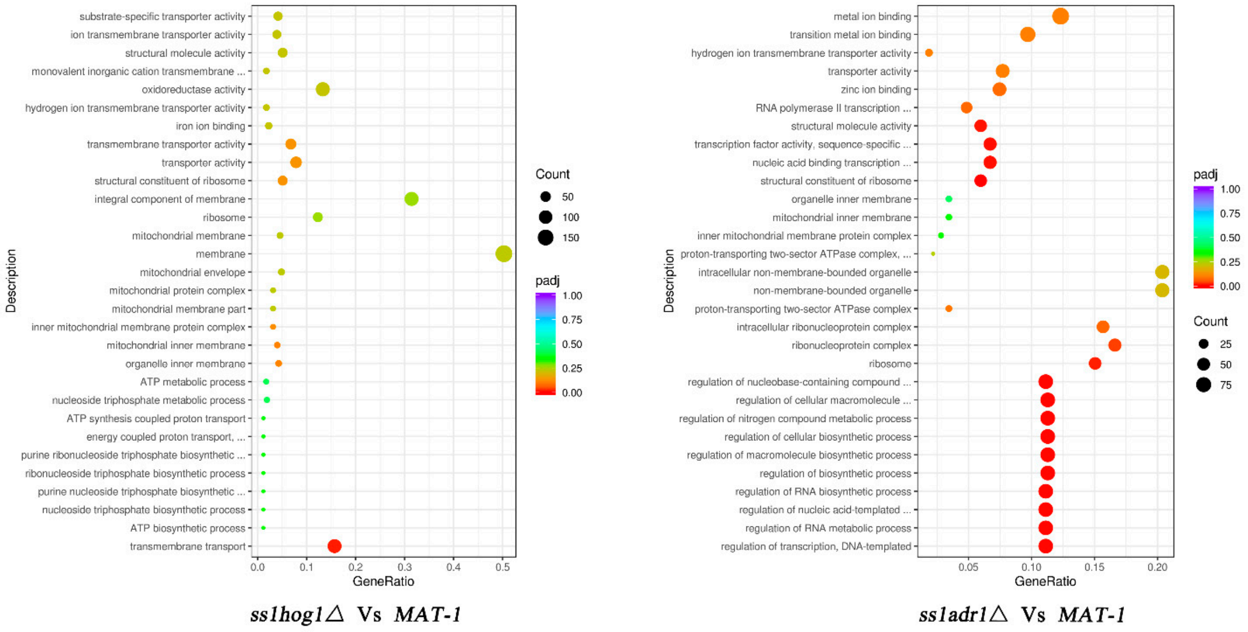

Figure S2. GO enrichment analysis of differentially expressed genes (DEGs) in the *MAT-1*, *ss1hog1*Δ, and *ss1adr1*Δ strains. Figure 6. GO enrichment analysis of differentially expressed genes (DEGs) in the *MAT-1*, *ss1hog1*Δ, and *ss1adr1*Δ strains. The haploid sporidia was allowed to grow on YePSA medium at 28 °C for 4 days, and then the total RNAs of *S. scitamineum* was extracted with TRIzol reagent. The bubble charts illustrate the terms in which the DEGs were enriched. The enrichment factor was calculated by (the number of different genes in a term/total number of different genes in a term) / (total number of genes in a term/total number of genes in a database). A *p*-value < 0.05 was considered statistically significant.

Table S1. The primers and sequences used in this study.

The primers	Sequence (5′- - 3′)
	The primers for gene deletion
ZEO-LB-F	GATAGTTTAAACTGAAGGCGGG
ZEO-LB-R	GAAGTGCACGCAGTTGCCG
ZEO-RB-F	CAAGAACAAGCGCTGTCGCC
ZEO-RB-R	AGCGGGCAGTTCGGTTTCA
SsGPA3-LB-F	GAGGCAAGCCAAGCCAGTTGA
SsGPA3-LB-R	CCCGCCTTCAGTTTAAACTATCTTTGGGTGTGTGTGAGAGGCT
SsGPA3-RB-F	TGAAACCGAACTGCCCGCTTCGGCCAAGAAGCAGCGAT
SsGPA3-RB-R	AGAAGCGATCAGCAAGCAAGCA
SsUAC1-LB-F	ATGCAATCTGCACTTCGGCC
SsUAC1-LB-R	CCCGCCTTCAGTTTAAACTATCCTTCTTTGGGTTGCTCTCGTGC
SsUAC1-RB-F	TGAAACCGAACTGCCCGCTATGGTGGCCAGATCCTGGCTA
SsUAC1-RB-R	AGGAAGCAGATCCACCAGTCG
SsADR1-LB-F	CTTGTTGAGAGGTGGGCGAT
SsADR1-LB-R	CCCGCCTTCAGTTTAAACTATCGAGTTGGCGTGATGAGACGG
SsADR1-RB-F	TGAAACCGAACTGCCCGCTAGAGGAGGATTTGCGAGCG
SsADR1-RB-R	GATGAGCGTGGATGCGTTTG
	The primers for gene detection
SsGPA3-inside-F	ATGGGAAACTGTCTTTCTTCCACAGAC
SsGPA3-inside-R	TCACAGAATACCACTATCCTTGAGCG
SsUAC1-inside-F	CCGGCACCTCAGCCATCCTT
SsUAC1-inside-R	AGGTTGTCGTGCTTGCCGAG
SsADR1-inside-F	CAACAACACCGCCGTCCAAG
SsADR1-inside-R	CGTCACGGTCGGCAAATACG

SsGPA3-outside-F	GGCCGCCTCGATACTTCAGAG
SsGPA3-outside-R	TGCGGCGCACAGTAAGGTT
SsUAC1-outside-F	GAGACGCGGTTAAGCGGGAA
SsUAC1-outside-R	GGATCGGTCCAGGTTCTTCGG
SsADR1-outside-F	GCCGGAAAGGTAGATGCCCA
SsADR1-outside-R	TCATCCTCTTCGCCATCCACG
The primers for RT-qPCR	
qRT-GAPDH-F	CAGCTCGATGAAGGTCAAGAT
qRT-GAPDH-R	CACATCTGCTGGAAGGTAGAG
qRT-SsGPA3-F	CAAGTACATTCTCTGGCGTTTC
qRT-SsGPA3-R	AGTCGGATGTTGCTCGTATC
qRT-SsUAC1-F	GCACGACAACCTGAGTGTAA
qRT-SsUAC1-R	GCCGTCAAAGAGACCAAAGA
qRT-SsADR1-F	CGTGCTGCTCTACGAAATGC
qRT-SsADR-R	AAGATCCTTGACGCCCGTTT
qRT-SsHOG1-F	AGTGGACGTACTTGAGACCT
qRT-SsHOG1-R	ATCTCGCCTCTTGAGGACAT
qRT-CDR99456.1-F	ACTCTGCGTTGGTCATCTTT
qRT-CDR99456.1-R	ACCACCCAACCCTTGAATAC
qRT-CDS01502.1-F	GTTCTTCCGTCATCGGTTCTC
qRT-CDS01502.1-R	GAGCCCTTGTTGAGCTTCTT
qRT-CDU25217.1-F	CCGCTGCTACTGGATATTTCA
qRT-CDU25217.1-R	CTTGATGCTGAGAGGGTAAGTC
qRT-CDS00122.1-F	CAGACACACCACAATCGGTAT
qRT-CDS00122.1-R	GATCATCGCCTGGGTAGAAAG
qRT-CDR88142.1-F	ACAAGACCGACGCCTACTGC
qRT-CDR88142.1-R	GATCTCACCCGAACGAGGCC
qRT-CDU25158.1-F	GGCACCAACGGCTACTTTGC
qRT-CDU25158.1-R	ACTGCCATGCCTCGCTTCTT
qRT-CDU24651.1-F	AGCGCAGGCTCTTTGTATT
qRT-CDU24651.1-R	TGGTTAAGCGTCCTGATGTG
